# Second‐To‐Fourth Digit Ratio (2D:4D) in Breast Cancer: A Systematic Review

**DOI:** 10.1002/ajhb.70321

**Published:** 2026-08-07

**Authors:** Abdullah Avcı, Emine Kaplan Serin

**Affiliations:** ^1^ Anesthesia Program, Vocational School of Health Services Toros University Mersin Turkey; ^2^ Department of Medical Nursing, Faculty of Nursing Mersin University Mersin Turkey

**Keywords:** 2D:4D, breast cancer, digit ratio

## Abstract

**Introduction:**

The second‐to‐fourth digit ratio (2D:4D) is considered an indirect marker of prenatal sex hormone exposure and has been investigated as a potential indicator of susceptibility to hormone‐related diseases, including breast cancer. The purpose of this systematic review is to evaluate the relationship between the 2D:4D finger ratio and breast cancer risk.

**Methods:**

This systematic review was conducted in accordance with the PRISMA 2020 statement. PubMed, Scopus, and Web of Science were searched without publication year restrictions. Eligible observational studies were selected using predefined PICOS criteria, and methodological quality was assessed using Joanna Briggs Institute critical appraisal checklists.

**Results:**

Seven observational studies involving 13 556 women (2817 breast cancer cases and 10 739 controls) were included. Most case–control studies reported significantly higher right‐ and/or left‐hand 2D:4D ratios among women with breast cancer than among healthy controls. However, the prospective cohort study reported an association only for the left‐hand 2D:4D ratio, whereas the largest multicenter case–control study did not demonstrate a consistent overall association between right‐ or left‐hand 2D:4D ratios and breast cancer risk. Overall, current findings suggest a possible association between higher 2D:4D ratios and breast cancer risk, although substantial heterogeneity exists across studies.

**Conclusion:**

This systematic review suggests that the 2D:4D finger ratio may be associated with breast cancer risk. However, substantial heterogeneity across studies warrants cautious interpretation, and further large prospective studies are needed to clarify its potential role in breast cancer risk assessment.

## 1 Introduction

Breast cancer is the most common type of cancer in women. According to GLOBOCAN 2022 data, breast cancer ranks second in global cancer incidence, accounting for 11.6% of all cancer cases with an estimated 2.3 million new cases. In the same year, 666 000 deaths occurred due to breast cancer, accounting for 6.9% of global cancer deaths and placing breast cancer fourth in cancer mortality (Bray et al. 2024). Despite early diagnosis opportunities, advanced treatment methods, and increased survival rates, breast cancer affects an individual's life in multiple ways: physically, psychologically, and socially throughout the diagnosis and treatment process and beyond (Culbertson et al. 2020).

The ratio between the lengths of the second (2D; index finger) and fourth (4D; ring finger) digits, commonly referred to as the 2D:4D ratio, is thought to be established during early prenatal development and to remain relatively stable throughout life (Manning et al. 1998; Manning 2011). Experimental studies suggest that this ratio is influenced by the balance between prenatal androgen and estrogen signaling during a critical period of fetal digit development. Androgen receptor (AR) and estrogen receptor alpha (ER‐α) expression are greater in the developing fourth digit than in the second digit. Increased androgen signaling promotes the growth of the fourth digit, resulting in a lower 2D:4D ratio, whereas increased estrogen signaling is associated with relatively reduced growth of the fourth digit and a higher 2D:4D ratio. These hormonal influences act during a narrow developmental window and are thought to produce a lifelong phenotypic marker of prenatal hormonal exposure (Zheng and Cohn 2011). While a lower 2D:4D ratio is generally observed in men, this ratio tends to be higher in women. This sexual dimorphism is thought to reflect differences in prenatal androgen and estrogen exposure. Because prenatal hormonal conditions may influence the development of multiple organ systems and contribute to disease susceptibility later in life, the 2D:4D ratio has been proposed as an indirect and non‐invasive marker of prenatal hormonal influences that may be relevant to adult health outcomes (Manning et al. 1998; Manning 2011; Grotmol et al. 2006).

Reproductive hormones are known to play an important role in the development and progression of breast cancer, a hormone‐dependent malignancy (Folkerd and Dowsett 2010). In addition, there is growing interest in the possibility that hormonal conditions during prenatal development may contribute to breast cancer susceptibility later in life, although the available evidence remains limited and is still evolving (Grotmol et al. 2006).

The 2D:4D ratio has been widely investigated as a putative marker of prenatal hormonal exposure. Previous studies have examined its associations with a range of physiological and health‐related outcomes, including reproductive function, cardiovascular health, physical performance, and certain neuropsychiatric traits (Elvan et al. 2025; Gower et al. 2025; Guo and Li 2025; Klimek et al. 2016). These associations have contributed to interest in the potential role of the 2D:4D ratio as a marker of developmental influences on adult health. However, evidence regarding its relationship with hormone‐related cancers, particularly breast cancer, remains limited and inconsistent (Bunevicius 2018; Kouam et al. 2024).

Although systematic reviews and meta‐analyses have previously linked the 2D:4D ratio to the risk of developing various types of cancer and the level of malignancy (Bunevicius 2018; Fonseca et al. 2022), there is no comprehensive systematic review addressing this relationship specifically in the context of breast cancer. Therefore, the aim of this study is to systematically evaluate the possible relationship between the 2D:4D finger ratio and breast cancer. Although the 2D:4D ratio has been proposed as a potential marker of prenatal hormonal exposure and disease susceptibility, its clinical utility as a screening or diagnostic tool for breast cancer has not yet been established. Nevertheless, clarifying the relationship between the 2D:4D ratio and breast cancer may contribute to future research aimed at improving risk assessment strategies and understanding the developmental origins of breast cancer.

## 2 Methods

This study was designed as a systematic review. The systematic review protocol was developed in accordance with the PRISMA‐P (Preferred Reporting Items for Systematic Review and Meta‐Analysis Protocols) guideline, and the reporting of the study was based on the PRISMA 2020 statement (Page et al. 2021). The systematic review protocol has been registered in the PROSPERO international systematic review registration platform (CRD420251103853).

A comprehensive literature search was conducted in PubMed, Scopus, and Web of Science using the Advanced Search functions of each database. In PubMed, the search was performed in the “All Fields” category using the expression “(2D:4D ratio OR digit ratio) AND breast cancer”. In Scopus, the search was conducted within the title, abstract, and keywords fields using the query “TITLE‐ABS‐KEY (2D:4D ratio OR digit ratio) AND TITLE‐ABS‐KEY (breast cancer).” In Web of Science, the topic field was searched using the expression “TS = (2D:4D ratio OR digit ratio) AND TS = (breast cancer).” The same core search terms and Boolean operators (AND, OR) were applied across all databases, and no restrictions were imposed on publication year. The PICOS framework was used to guide eligibility assessment and study selection. In addition, the reference lists of eligible studies were manually screened to identify potentially relevant publications not retrieved through the electronic database searches.

**FIGURE 1:**
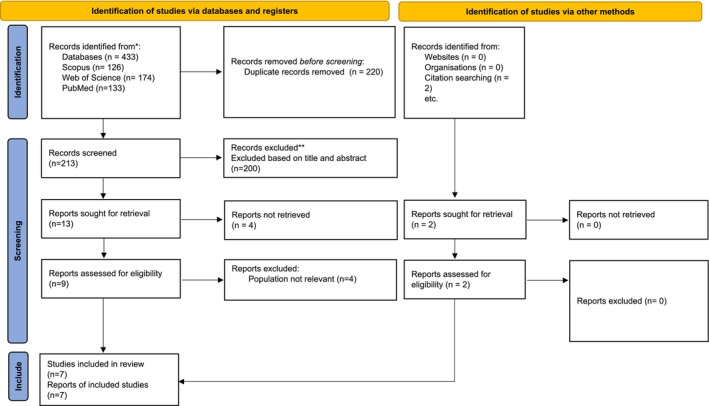
PRISMA 2020 flow diagram for new systematic reviews, which included searches of databases, registers, and other sources.

The literature review was conducted independently by two researchers (A.A. and E.K.S.). The process of accessing the seven studies included in the systematic review from a total of 433 studies is presented in the PRISMA 2020 flow diagram, categorized according to databases (Figure 1).

The selection of studies to be included in this systematic review was based on inclusion and exclusion criteria determined according to the PICOS (P = Population, I = Intervention/Exposure, C = Comparison, O = Outcome, S = Study design) framework (Table 1).

**TABLE 1:** Inclusion and exclusion criteria for the studies.

PICOS	Inclusion criteria	Exclusion criteria
P (Population)	Adult women aged ≥ 18 years with a diagnosis of breast cancer and healthy female controls evaluated for comparison purposes	Studies including only children or adolescents; animal studies; individuals with cancers other than breast cancer
I (Indicator/Exposure)	Assessment of second‐to‐fourth digit ratio (2D:4D)	Studies not measuring 2D:4D ratio
C (Comparison)	Healthy control group and/or comparison across breast cancer pathological subtypes	Studies without a comparison group or subtype‐based analysis
O (Outcome)	Differences in 2D:4D ratios between breast cancer patients and controls and/or across pathological subtypes	Studies measuring 2D:4D ratios only in individuals without breast cancer
S (Study design)	Observational studies (descriptive, cross‐sectional, case–control, cohort, prospective or retrospective) published in English with accessible full text	Reviews, meta‐analyses, abstracts, conference proceedings, commentaries, qualitative studies, case reports, randomized controlled trials, and studies with inaccessible full text or not published in English

During the selection process, titles and abstracts were first reviewed, and the full texts of suitable studies were evaluated in detail. A standard data extraction form was developed by researchers for the systematic summarization of data, and all data were collected using this form. Using the data extraction form, the following details of the studies included in the systematic review were recorded: author(s), year of publication, country where the study was conducted, study design, sample size, mean age, characteristics related to the measurement of the 2D:4D finger ratio, and measurement methods used. The data extraction process was performed independently by two researchers, and the data obtained were then compared and jointly verified. In cases where researchers disagreed, consensus was reached based on the literature.

The methodological quality of the studies included in this systematic review was assessed using critical appraisal checklists developed by the Joanna Briggs Institute (JBI) in accordance with the study designs. The JBI Critical Appraisal Checklist for Case Control Studies was used for studies with a case–control design, while the JBI Critical Appraisal Checklist for Cohort Studies was used for studies with a prospective cohort design. Both checklists consist of structured questions where items are evaluated using the options “Yes,” “No,” “Uncertain,” or “Not Applicable.” The evaluation process was conducted independently by two researchers (A.A., E.K.S.); in case of disagreement, consensus was reached based on the literature. The total number of “Yes” responses given for each study was used to indicate the methodological quality level of the study. It has been determined that methodological quality scores range from 7/10 to 12/12. While quality scores in case–control studies ranged from 7/10 to 10/10, the prospective cohort study was rated 12/12 (Tufanaru et al. 2020).

A total of 433 studies were identified through database searches in this systematic review. 220 of these studies were excluded due to duplication. As a result of the title and abstract review, 200 studies were excluded. Four of the nine studies included in the full‐text review were excluded because they did not meet the inclusion and exclusion criteria. Following this stage, the citation search process for the five studies included in the systematic review has been initiated. In this regard, the reference lists of the studies were scanned, and the full texts of four studies were examined for research suitability. Two of these records met the inclusion criteria for the study. Seven studies have been identified that examine the possible relationship between the 2D:4D finger ratio in patients with breast cancer. The PRISMA flow diagram illustrating the selection process of the studies is shown in Figure 1.

## 3 Results

This systematic review included a total of seven observational studies examining the relationship between the second and fourth finger ratio (2D:4D) and breast cancer. Most of the studies were conducted using a case–control design, with only one study employing a prospective cohort design. The included studies were published between 2012 and 2025 and included samples from different geographical regions such as Turkey, India, Brazil, Serbia, China, and Australia. In case–control studies, breast cancer diagnosis was histopathologically confirmed, and participants consisted of women aged 18 years and older. Controls were generally selected from age‐matched healthy women and included individuals with no history of breast cancer or hormone‐related malignancy. Some studies have also evaluated menopausal status and pathological subtypes of breast cancer (Luminal A, Luminal B, HER2‐overexpressing, and triple‐negative).

In the included studies, the second‐to‐fourth finger ratio (2D:4D) was calculated by dividing the length of the index finger (2D) by the length of the ring finger (4D). Both direct and indirect measurement methods were employed. Studies using direct measurement methods measured finger lengths with Vernier calipers (Jafar et al. 2018; Udicki et al. 2023; Moirangthem et al. 2025), digital Vernier calipers (Mendes et al. 2016), or a combination of photocopy and Vernier caliper (Muller et al. 2012). Measurements were generally performed with an accuracy of 0.01 mm and taken from the fingertip to the most proximal point of the palmar digital crease, separately for the right and left hands. Studies using indirect measurement methods used digital scanning (Dişli et al. 2024) or photocopying (Hong et al. 2014) followed by measurement with an electronic Vernier caliper. In these studies, measurements were mostly repeated twice, and average values were used in the analyses.

Among the included case–control studies, most investigations (Hong et al. 2014; Jafar et al. 2018; Mendes et al. 2016; Udicki et al. 2023) reported higher 2D:4D ratios in women with breast cancer compared with controls, although the affected hand differed across studies. In contrast, the large multicenter study by Moirangthem et al. (2025) found no consistent overall association between digit ratios and breast cancer risk. Hong et al. observed significantly higher bilateral digit ratios in patients, with the strongest difference in the left hand, whereas Jafar et al. reported significantly elevated right‐ and left‐hand ratios in breast cancer patients (both *p* < 0.0001). Similarly, Mendes et al. found higher right 2D:4D, left 2D:4D, and Δ*r* − l values among cases than controls. Udicki et al. detected a significant difference only for the right‐hand ratio and identified a cut‐off value of 1.012 for predicting breast cancer development. However, it should be noted that the breast cancer group in this study was considerably older than the control group (59.0 ± 10.8 vs. 39.8 ± 11.8 years), which may have introduced residual confounding and should be considered when interpreting these findings. In contrast, the prospective cohort study by Muller et al. (2012), which included 9044 women and 573 incident breast cancer cases, demonstrated no association between right 2D:4D and breast cancer risk (HR = 1.01, 95% CI 0.93–1.10), whereas higher left 2D:4D was associated with increased risk (HR = 1.12, 95% CI 1.02–1.21). Moreover, a higher Δ*r* − l was associated with a lower breast cancer risk (HR = 0.90, 95% CI 0.83–0.98). Regarding age at diagnosis, Mendes et al. reported an inverse correlation between left‐hand 2D:4D and age at breast cancer diagnosis (r = −0.246, *p* = 0.014), while Udicki et al. found a weak negative association for right‐hand 2D:4D (*r* = −0.271, *p* < 0.05). Hong et al. similarly demonstrated significant negative associations between digit ratios and age among breast cancer patients. In the cohort study, Muller et al. observed that increasing right 2D:4D and Δ*r* − l values were associated with younger age at diagnosis. Furthermore, Muller et al. reported no relationship between digit ratios and age at menarche but found that higher right 2D:4D and Δ*r* − l values were associated with earlier menopause. In a recent multicenter case–control study from India involving 1638 breast cancer patients and 1723 controls, Moirangthem et al. (2025) reported no consistent overall association between right‐ or left‐hand 2D:4D ratios and breast cancer risk. However, subgroup analyses showed a significantly lower breast cancer risk among premenopausal women in the highest quintile of right‐hand 2D:4D (OR = 0.71, 95% CI 0.52–0.96), whereas a non‐significant positive association was observed among postmenopausal women. In addition, increasing Δ*r* − l values were associated with a reduced breast cancer risk (OR = 0.81, 95% CI 0.68–0.95), particularly among premenopausal women. Correlations between age at diagnosis and right‐hand 2D:4D or Δ*r* − l were weak and non‐significant. Another study by Dişli et al. (2024), including 159 breast cancer patients and 50 healthy controls, primarily investigated differences according to molecular subtypes (luminal A, luminal B, HER2‐overexpressing, and triple‐negative breast cancer). However, no statistically significant difference was observed between groups regarding age, and the available results provided limited evidence for a clear association between digit ratio and breast cancer subtype. The characteristics of the included studies, including country of origin, study design, sample size, participant age, digit measurement methods, main findings regarding the association between 2D:4D ratio and breast cancer, and methodological quality scores, are summarized in Table 2.

**TABLE 2:** Characteristics of the included studies.

First author, year (reference)	Country of study	Study design	Sample size (*n*)		Mean age (SD)		Digit measurement method	Correlations between 2D:4D and breast cancer	Quality score
			Cases	Controls	Cases	Controls			
Hong, 2014	China	A case–control study	109	109	46.7 (4.9)	47.4 (4.9)	Photocopy + Electronic vernier caliper	In breast cancer patients, the 2D:4D ratio was found to be higher in both the right and left hands compared to controls, and in both groups, the 2D:4D ratio in the left hand was higher than in the right hand.	7/10
Jafar, 2018	India	A case–control study	145	145	Unreported	Unreported	Vernier caliper	The 2D:4D ratio is significantly higher in breast cancer cases compared to controls.	7/10
Muller, 2012	Australia	A prospective cohort study	573	8471	55 (?)	53 (?)	Photocopy + Vernier caliper	The left hand 2D:4D ratio was found to be positively associated with breast cancer risk; the right hand 2D:4D ratio was not associated with risk, and an inverse relationship was found between the right–left difference and risk.	11/12
Udicki, 2023	Serbia	A case–control study	98	141	59.0 (10.8)	39.8 (11.8)	Vernier caliper	In breast cancer patients, the right hand 2D:4D ratio is significantly higher compared to healthy controls.	7/10
Moirangthem, 2025	India	A case–control study	1638	1723	48.20 (7.73)	47.29 (7.10)	Vernier caliper	An inverse association was found between the right–left difference in 2D:4D ratio and breast cancer risk in premenopausal women.	10/10
Dişli, 2024	Turkey	A case–control study	154	50	a: 48.8 (14.0)	50.3 (10.6)	Digital scanning	The study found that the right hand 2D:4D ratio was significantly lower in the Luminal A breast cancer group compared to other subgroups.	8/10
					b: 51.3 (10.3)				
					c: 55.4 (13.0)				
					d: 52 (11.4)				
Mendes, 2016	Brazil	A case–control study	100	100	55.63 (11.60)	55.4 (11.5)	Digital vernier caliper	In breast cancer patients, the right and left hand 2D:4D ratios and the right–left difference are higher than in controls.	10/10

*Note:* a: Triple Negative, b: Luminal B, c: Luminal A, d: HER2over‐expressing.

## 4 Discussion

In the literature, there are various systematic review and meta‐analysis studies examining the relationship between the 2D:4D finger ratio and cancer risk (Bunevicius 2018; Fonseca et al. 2022). However, these studies have generally addressed cancer types collectively. This situation indicates a gap in the literature regarding the specific and systematic examination of the relationship between breast cancer and the 2D:4D ratio. This systematic review comprehensively examined studies investigating the possible relationship between the 2D:4D ratio and disease in individuals diagnosed with breast cancer, aiming to fill this gap. In this regard, a comprehensive literature review was conducted and seven studies meeting the inclusion criteria were analyzed. However, these studies show significant heterogeneity in terms of sample size, study design, and finger length measurement methods. These methodological differences limit the generalizability of the findings and require careful interpretation.

The inconsistency among studies may be explained by differences in ethnicity, sample size, menopausal status, study design, measurement techniques (direct measurements, photocopies, or digital scans), and adjustment for potential confounding factors. These methodological variations may partly account for the heterogeneous findings reported across studies. The observed association between higher 2D:4D ratios and breast cancer risk may reflect the influence of prenatal sex steroid exposure on mammary gland development and subsequent susceptibility to breast carcinogenesis (Ventura et al. 2013; Soto et al. 2013). Interestingly, the subtype‐specific differences observed in digit ratio, particularly in Luminal A tumors, may suggest that prenatal hormonal influences vary according to breast cancer subtype. Given that Luminal A tumors are strongly hormone‐dependent and characterized by estrogen receptor positivity, prenatal endocrine factors may contribute differently to subtype‐specific tumor biology, although current evidence remains limited and should be interpreted cautiously. The 2D:4D ratio is considered an indirect marker of the balance between prenatal androgen and estrogen exposure (Manning et al. 1998; Zheng and Cohn 2011). Prenatal hormonal milieu may influence mammary gland organization, endocrine responsiveness, and susceptibility to later‐life carcinogenic stimuli. Consequently, digit ratio may reflect long‐term developmental programming rather than adult hormonal exposure alone (Diaz‐Santana et al. 2020; Soto et al. 2013). The heterogeneous associations reported across different malignancies suggest that the biological significance of 2D:4D is likely cancer‐specific rather than universal (Bunevicius 2018; Fonseca et al. 2022). Bunevicius (2018) reported that low digit ratios were more frequently associated with prostate and brain cancers, whereas breast cancer tended to be associated with higher ratios.

Interestingly, several included studies (Hong et al. 2014; Mendes et al. 2016; Udicki et al. 2023) reported an inverse association between the 2D:4D ratio and age at diagnosis. Similar findings were summarized by Bunevicius (2018), who reported that higher 2D:4D ratios were associated with younger presentation of breast cancer. Comparable inverse associations between digit ratio and age at disease presentation have also been observed in non‐malignant conditions such as coronary heart disease (Lu et al. 2015), suggesting that prenatal hormonal influences may affect not only disease susceptibility but also the timing of clinical manifestation. These observations raise the possibility that prenatal hormonal factors, indirectly reflected by digit ratio, may influence not only breast cancer susceptibility but also the age at disease onset. Although the biological mechanisms underlying these findings remain incompletely understood, the available evidence supports the hypothesis that early developmental and endocrine influences may contribute to breast carcinogenesis.

Although the 2D:4D ratio has attracted interest as a potential anthropometric marker, the current evidence mainly emphasizes its value for understanding the developmental and hormonal mechanisms underlying breast carcinogenesis rather than its application as a diagnostic tool.

## 5 Recommendations for Future Research

Future studies should be conducted with larger and more representative samples, particularly those with a prospective cohort design. This allows for a more robust assessment of the potential causal relationship between the 2D:4D ratio and breast cancer. Standardizing measurement techniques (e.g., measuring using a digital caliper based on specific anatomical reference points) will improve data quality. Additionally, systematically evaluating hand preference (right/left) will contribute to understanding the relationship between 2D:4D lateralization and disease risk. In addition, future studies should correlate the 2D:4D ratio not only with cancer development but also with clinical variables such as tumor subtypes, stage, hormone receptor status, and prognosis; thus, the effects of prenatal hormone exposure on cancer biology should be more comprehensively elucidated. Finally, there is also a need for multicenter studies evaluating the usability of the 2D:4D ratio in screening and early diagnosis processes.

## 6 Conclusion

This systematic review has compiled current evidence evaluating the possible relationship between the ratio of the lengths of the second and fourth fingers (2D:4D) and breast cancer. The findings reveal that breast cancer patients generally have higher 2D:4D ratios compared to healthy controls. The results obtained suggest that prenatal hormone exposure may play a role in the development of breast cancer in adulthood. However, due to methodological differences in the studies included in the systematic review, diversity in measurement techniques, and sample differences, caution should be exercised in interpreting the findings. Therefore, large‐scale, prospective studies using standardized measurement methods are needed to more clearly demonstrate the future applicability of the 2D:4D ratio in predicting breast cancer risk.

## 7 Limitations

This systematic review should be evaluated with certain limitations in mind. First, most of the included studies were conducted using a case–control design, which does not allow for establishing a causal relationship. There are significant differences in the sample sizes of the studies, and the fact that some studies were conducted with small samples limits the generalizability of the results. The variety of measurement methods (the use of different tools such as digital calipers, photocopiers, and digital rulers) increases the risk of systematic error between measurements. Furthermore, most studies did not provide information on which reference points were used for finger length measurements or on the intra‐ or inter‐observer reliability of the measurements. However, it is known that finger ratios can vary depending on genetic, ethnic, and environmental factors. Since the studies included in the compilation came from different countries, it has not been clearly controlled how these factors play a role in the relationship between 2D:4D and breast cancer. Additionally, some studies have focused solely on right or left hand measurements, while others have based their findings on average values. This situation has made comparative analysis difficult. An additional limitation of this review is that a meta‐analysis could not be performed. The included studies showed substantial methodological heterogeneity in terms of study design, participant characteristics, digit ratio measurement methods, outcome definitions, and reporting formats. Moreover, several studies did not provide sufficient quantitative data required for calculating pooled effect sizes.

## Author Contributions

**Abdullah Avcı** writing – review and editing, writing – original draft, supervision, methodology, investigation, data curation, conceptualization. **Emine Kaplan Serin:** writing – review and editing, writing – original draft, methodology, investigation, conceptualization.

## Funding

The authors have nothing to report.

## Ethics Statement

This research involved secondary data analysis and was therefore not performed on human or animal subjects.

## Conflicts of Interest

The authors declare no conflicts of interest.

## Data Availability

The data that support the findings of this study are available from the corresponding author upon reasonable request.
